# Low-Temperature Continuous Flow Synthesis of Metal Ammonium Phosphates

**DOI:** 10.1038/s41598-018-31694-x

**Published:** 2018-09-10

**Authors:** Alistair F. Holdsworth, Harry Eccles, Alice M. Halman, Runjie Mao, Gary Bond

**Affiliations:** 1 Division of Chemistry, School of Physical Sciences and Computing, University of Central Lancashire, Preston, United Kingdom; 2 Department of Engineering, University of Liverpool, Liverpool, United Kingdom

## Abstract

The synthesis of the high performance inorganic materials essential to the quality of modern day life is hindered by traditionalist attitudes and reliance on outdated methods such as batch syntheses. While continuous flow methods have been extensively adopted in pharmaceutical circles, they remain largely unexplored for the preparation of inorganic compounds, despite higher efficiency, safety and versatility. In this publication, we demonstrate a step-change for the synthesis of metal ammonium phosphates through conversion of the extant batch process to a low-temperature continuous regime, exhibiting a tenfold increase in throughput combined with a significant decrease in particle size.

## Introduction

Although inorganic materials are essential to life in the 21^st^ century, advancements in their preparation are underutilised as traditionalist attitudes in academia and particularly industry mean batch processes predominate. Continuous flow synthesis (CFS) is one such alternative technique: it is prevalent in pharmaceutical research^1,2^, yet has received lesser attention for inorganic synthesis outside of nanomaterials research^3^. This is largely due to the typically high capital costs associated with flow systems^4^; despite the technique’s inherent flexibility^1,2,4^, safety^1,2^, scalability^1,3,5,6^, and controllability^1–3,7,8^. Low temperature (<100 °C), ambient pressure flow syntheses presents an affordable preparative route to a plethora of inorganic materials^8–15^, without the high capital cost of proprietary systems^4^. In this communication, we demonstrate a step-change, low-cost continuous preparation of versatile metal ammonium phosphates and derivatives. We compare CFS with the commonly used batch method^16^ and demonstrate a tenfold increase in throughput combined with a significant reduction in particle size for continuously synthesised particles.

Metal ammonium phosphates (MAPs) and other compounds of the form AMPO_4_.xH_2_O (where A^+^ is Na, K, NH_4_ and M^2+^ is a divalent metal such as Mg, Fe or Zn) were first explored in detail by Bassett and Bedwell in the 1930s^16^. These compounds have since been investigated for a myriad of uses including catalysis^17–19^, pigments^17^, fertilisers^20^, energy storage^21^, biomaterials^8–11^, flame retardants^17^ and as ion exchange materials^22–28^. Their preparative route, however, has changed little since the initial research^16^: an inefficient batch precipitation process requiring vast excesses of phosphorus reagent, and extended reaction times (>3 h) at high temperatures (>80 °C) to fully crystallise the product. Hydrothermal and solvothermal routes to MAPs and related compounds have been explored^29–33^, though these are often as, if not more time, resource and energy intensive than the traditional precipitation route.

Based upon a novel low temperature (<100 °C) ambient pressure continuous flow synthesis of the biomaterials brushite and hydroxyapatite^8–11^, we adapted the synthesis of MAPs to a continuous mode. Bassett noted that an additional source of NH_4_^+^ or performing the precipitation at an elevated pH, can serve to increase the rate of crystallisation of some MAPs^16^. Through a combination of these factors and the rapid mixing of a continuous flow regime, we are able to reduce the reaction time for preparation of MAPs from a typical 180 minutes down to 7 minutes. An excess of phosphate relative to the metal is required to prevent formation of the undesired M_3_(PO_4_)_2_ phase^16,34^.

We prepared six MAPs (M = Mg, Mn, Fe, Co, Ni and Zn) and one related hydrogen phosphate (M = Sn) using this technique in a variety of hydration states. Our CFS process provides two feeds: one containing the metal (M^2+^, 0.05 M) typically as the nitrate salt, and the other a mixture of triammonium phosphate (TAP, 0.25 M) and ammonium nitrate (AN, 0.5 M)^8–11^. These feeds mix continuously before heating the flow, which serves to crystallise the initially amorphous product^8^. For some metals (notably Fe and Co), this crystallisation results in a clear colour change. The characterisation data of our MAPs are presented in Table 1, with comparative particle sizes, yields, and throughputs for material produced using the common batch process^16^. Reaction yields are expressed as a percentage of the theoretical maximum. Throughputs are expressed in grams per litre of liquor per hour, accounting for the reaction time.

**Table 1:** Characterisation data for MAPs: reaction yield (%), particle size for flow (CFS) and batch (Bx) produced material as 10^th^, 50^th^ and 90^th^ percentiles (D_10_, D_50_ and D_90_, µm), BET surface are (m^2^/g) and observed XRD crystal hydration phase.

M	XRD Phase		Yield		Particle Size (CFS, um)			Particle Size (Bx, um)			Throughput		BET
	(CFS)	(Bx)	% (CFS)	% (Bx)	D_10_	D_50_	D_90_	D_10_	D_50_	D_90_	CFS	Bx	m^2^/g (CFS)
MgAP*	Mon/Hex	Mon	94	99	2.5	13.6	44.3	14.6	24.6	66.0	6.6	1.3	15.7
MnAP	Mon	Mon	99	99	6.4	22.8	65.1	7.4	18.4	42.2	8.4	1.5	6.8
FeAP	Mon	Mon	99	99	2.9	9.5	23.9	15.4	52.8	108.3	8.4	1.6	49.8
CoAP	Mon	Mon	97	99	4.9	23.5	51.3	15.8	65.0	135.9	8.4	1.6	6.5
NiAP	Hex	Mon	83	99	1.3	7.7	26.6	2.6	12.1	81.4	7.1	1.6	9.5
ZnAP	Anhyd	Anhyd	98	99	2.0	9.7	20.9	6.3	19.9	45.5	7.9	0.5	3.6
SnHP	Anhyd	Anhyd	79	78	2.7	6.5	22.8	2.3	19.4	40.3	7.5	1.5	4.7

MgAP (*) required the addition NH_3_ to the Mg^2+^ feed in flow to precipitate the desired product^8^.

**Figure 1:**
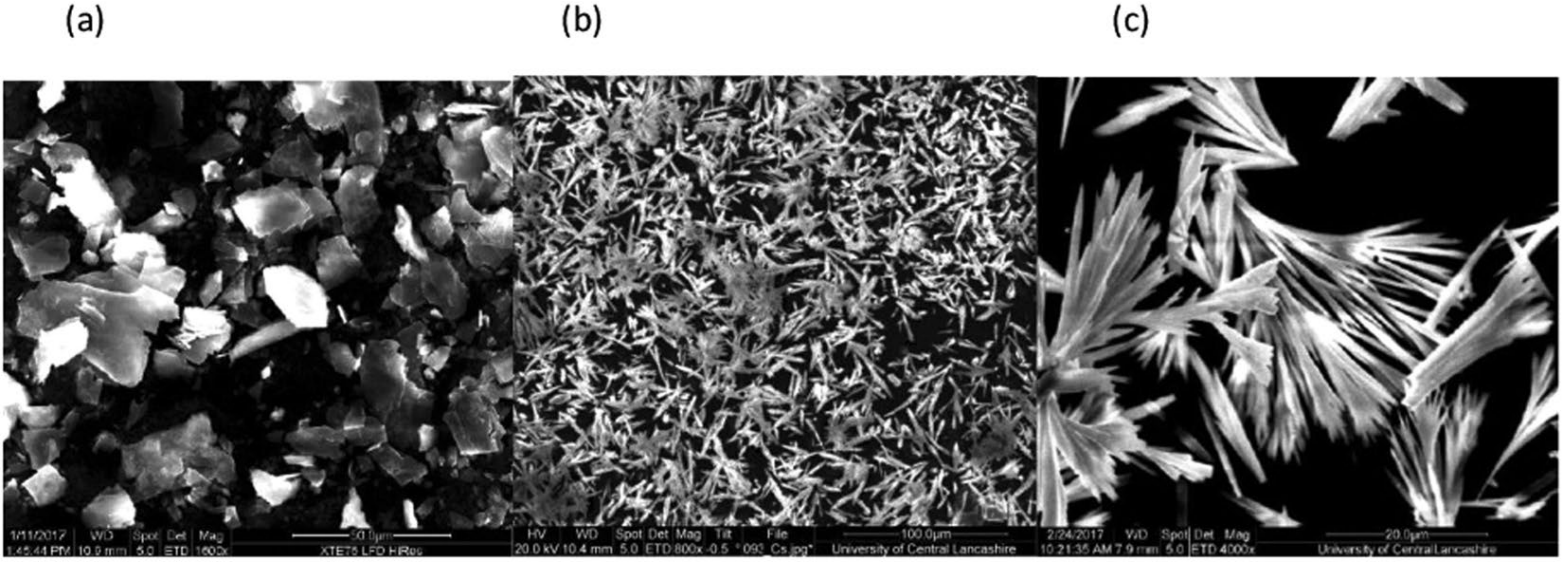
SEM images of FeAP platelets, ZnAP flowers and SnHP rods.

MgAP forms a mixed-phase mono- and hexa-hydrate in CFS, and a monohydrate in batch (Fig. S1)^19,35–37^. MnAP, FeAP and CoAP are all prepared in the monohydrate dittmarite phase regardless of synthesis method (Figs S2–S4)^19,35–37^, forming plate-like crystallites (Fig. 1a). NiAP forms the hexahydrate (Fig. S5) struvite-like phase in flow, and the monohydrate in batch^34^. ZnAP forms an anhydrous phase regardless of synthesis method (Fig. S6)^16^, forming flower-like crystallites (Fig. 1b). SnHP forms anhydrous rod-like particles (Figs 1c and S7).

MgAP can interchange between mono- and hexahydrated phases through hydrolysis and dehydration respectively^35,38^, though our continuous flow process forms a mix of the two phases, though a longer or hotter reaction time fully dehydrates the product. NH_3_ was added to the Mg^2+^ feed to prevent formation of MgHPO_4_^8^. Mn, Fe and CoAP all favour the monohydrated phase and were prepared in this form in both batch and flow^16,19,36,37^. NiAP can, like MgAP, interconvert between the two phases, though the monohydrate phase is metastable and the synthesis is sensitive to the reaction conditions used^34^. The NiAP batch sample was of the monohydrate phase as the crystals have sufficient time to dehydrate. No stable ZnAP hydrate has been prepared as the system favours the anhydrous form^16^. We were unable to synthesise SnAP as hydrolysis of the acidic Sn^2+^ ion takes precedence over the formation of the desired phase. SnHP is prepared by exchanging the triammonium phosphate for phosphoric acid and omitting the ammonium nitrate. The phases of the batch produced materials match those of the flow produced materials except for NiAP^34^, which is synthesised as the monohydrate in batch^16^. Neither CaAP or CuAP can be synthesised via our continuous flow route, as hydroxyapatite^39^ (for Ca) or a hydroxyphosphate^40^ (for Cu) form preferentially instead. Bassett noted the difficulty in synthesising CuAP relative to other divalent metals^16^.

The yields of all our flow process were excellent (>94%) except for NiAP (83%) and SnHP (79%) due to the marginal solubilities of the metals in aqueous ammonia and acid respectively^41–43^. The yields can be increased through process optimisation. Yields for the batch produced material were quantitative, except for SnHP, where the yield matched the flow produced example. All of our flow produced materials, except MnAP, were at least half the size than the batch produced materials with narrow size distributions. Flow-produced FeAP is less than one fifth the size of equivalent batch material. The reduction in particle size is due to the increased rates of mixing and turbulence within the flow process and our adapted reaction conditions which promote a significant reduction in crystallisation time. For MnAP, the larger sized particles produced in flow are due to increased Ostwald ripening during the crystallisation process^44^. The observed surface areas of our flow-produced compounds vary with the nature of the crystalline product. FeAP shows a BET high surface area as the plate-like crystals are significantly thinner (Fig. 1a) than those of the similarly plate-like MnAP and CoAP. Throughputs for the CFS process are at least five times that of the batch process, and up to sixteen times for ZnAP due to the lengthy reaction times required in batch^16^.

The efficiency of the CFS process can be further improved by the feasible regeneration of the effluent liquor. Concentration, replacement of precipitated phosphate and addition of the required NH_3_ allows for use in further syntheses. Recovery of process heat by pre-heating precursor streams would further increase efficiency. Changing reagent stoichiometry, overall concentrations, and process parameters such as temperature, flow rate and mixing regime would allow for control of particle sizes produced within the CFR^45^. Use of specialist mixers such as confined impinging jet mixers may also allow for preparation of MAPs on the nanoscale due to more turbulent mixing during the precipitation step^45^. The flows within our demonstrated system are all laminar.

## Conclusions

We have developed an efficient continuous flow synthesis of metal ammonium phosphates and compared this to traditional batch methods. We have demonstrated a step change in the preparation of these compounds as synthesised particles are smaller, more evenly sized and produced with far greater efficiency than previous syntheses. In comparison to the traditional batch methods, we observe up to a tenfold increase in throughput (in terms of mass per unit volume per unit time) with our flow process. This value can be increased further with process optimisation. For many applications, smaller particles are preferably as higher surface areas provide higher activities. Continuous flow synthesis provides this benefit with the option of greater control over particle size and morphology. Through the application of appropriate engineering and chemistry, the syntheses of other key inorganic materials could be improved in a similar manner as demonstrated here.

## Experimental

Mg(NO_3_)_2_, Mn(NO_3_)_2_, (NH_4_)_2_Fe(SO_4_)_2_, Co(NO_3_)_2_, Ni(NO_3_)_2_, Zn(NO_3_)_2_, SnCl_2_, NH_4_NO_3_, H_3_PO_4_, NH_4_OH, and (NH_4_)_2_HPO_4_ were obtained from Fisher Scientific as reagent grade (>98%) quality and used as acquired with no further purification. (NH_4_)_3_PO_4_ was prepared *in situ* by the addition of excess ammonia to a concentrated solution of (NH_4_)_2_HPO_4_ and gently heating the resultant mixture to 65 °C so as to remove the excess ammonia. The solution was then cooled and diluted to the required concentration. Deionised water (>18 MΩ/cm) was used for all syntheses.

**Figure 2:**
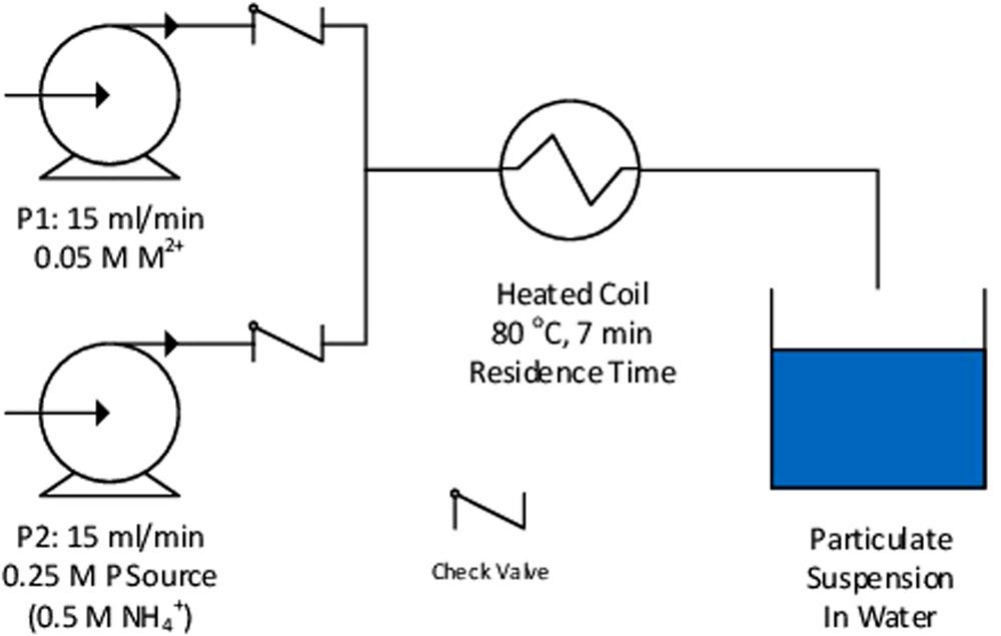
Schematic of CFR.

Our continuous flow reactor (CFR) was constructed based on previous literature references^8–11^. Simply, this consists of two peristaltic pumps (Watson Marlow SciQ 323), a simple Y-mixer and a coil of PVC tubing (16 m length, 4 mm internal diameter, wall thickness 1 mm, total volume 210 cm^3^) contained within a thermostatically-controlled water bath (Grant JB Aqua 18 Plus). This is illustrated in Fig. 2.

The rotational rate and thus flow rate of each pump can be varied from 5–40 ml/min, corresponding to a total flow rate of 10 to 80 ml/min and thus a residence time of between 3 and 20 minutes, though a total flow rate of 30 ml/min and thus a residence time of 7 minutes was used in this work. Both pumps were run at the same flow rate in all cases, with one providing the metal salt feed, and the other the phosphorus source and any ancillary reagents. The temperature of the water bath could be varied between ambient and 100 °C. All reactions presented here were performed at 80 °C. A narrow aperture (c.a. 1 mm) at the outlet of the CFR served to even the flow within the system, and check valves after each pump ensured a continuous unidirectional flow. Products were purified by repeated washing with deionised water, and were then dried overnight at 80 °C. The batch syntheses of MAPs were adapted from the work of Bassett and Bedwell^16^, using the same procedure but scaled to give the same volume of product as in our flow process.

The rate of heating within the process is rapid, as a total flow rate of greater than 120 ml/min emerges from the reactor at 80 °C with the same tubing setup utilised for these syntheses, suggesting that temperature equilibrium is attained within 2 minutes and thus 5 minutes are spent at temperature to allow completion of reaction. Further modelling of this factor is beyond the scope of the research presented here.

Powder X-Ray Diffraction (PXRD) analysis was conducted using a Brucker D2 Diffractometer with a copper kα radiation source with data collected between 5 and 80 degrees 2Θ. XRD patterns were converted from proprietary formats using PowDLL^46^. Scanning Electron Microscopy (SEM) and X-Ray Energy Dispersive elemental analysis was conducted under high vacuum using a FEI Quanta 200 scanning electron microscope equipped with an EDAX Sapphire Energy Dispersive X-Ray detector. Surface areas were determined using the Brunauer–Emmett–Teller (BET) model at 77 K using nitrogen absorption (Micrometrics ASAP2010), with accuracy checked against an alumina standard. Particle size distribution analysis (PSD) was conducted using a Malvern Mastersizer 2000. Samples were sonicated in deionised water before analysis to disperse any agglomerates. Results are expressed as D_50_, the 50^th^ percentile of particle size.

## Electronic supplementary material

Supplementary Figures

## Data Availability

The data underpinning this publication can be found on https://clok.uclan.ac.uk/.
