# Supplementary material for: Low-Temperature Continuous Flow Synthesis of Metal Ammonium Phosphates
Source: Sci Rep. 2018 Sep 10;8:13547. doi: 10.1038/s41598-018-31694-x (PMC6131346; doi:10.1038/s41598-018-31694-x)
Supplement: Supplementary file 1 — Supplementary Figures [file 41598_2018_31694_MOESM1_ESM.docx]

**Supplementary Figures**:

**Low-Temperature Continuous Flow Synthesis of Metal Ammonium Phosphates**

Alistair F. Holdsworth,^1,2^ Harry Eccles,^1*^ Alice M. Halman,^1^ Runjie Mao^1^ and Gary Bond^1^

**Figure S1:** XRD Patterns of batch (Bas) and flow (Fl) MgAP.

**Figure S2:** XRD Patterns of batch (Bas) and flow (Fl) MnAP.

**Figure S3:** XRD Patterns of batch (Bas) and flow (Fl) FeAP.

**Figure S4:** XRD Patterns of batch (Bas) and flow (Fl) CoAP.

**Figure S5:** XRD Patterns of batch (Bas) and flow (Fl) NiAP.

**Figure S6:** XRD Patterns of batch (Bas) and flow (Fl) ZnAP.

**Figure S7:** XRD Patterns of batch (Bas) and flow (Fl) SnHP.
